# Brexanolone, a GABA_A_ Modulator, in the Treatment of Postpartum Depression in Adults: A Comprehensive Review

**DOI:** 10.3389/fpsyt.2021.699740

**Published:** 2021-09-14

**Authors:** Amber N. Edinoff, Amira S. Odisho, Kendall Lewis, Amir Kaskas, Grace Hunt, Elyse M. Cornett, Alan D. Kaye, Adam Kaye, John Morgan, P. Scott Barrilleaux, David Lewis, Omar Viswanath, Ivan Urits

**Affiliations:** ^1^Department of Psychiatry and Behavioral Medicine, Louisiana State University Health Science Center Shreveport, Shreveport, LA, United States; ^2^Louisiana State University Shreveport School of Medicine, Shreveport, LA, United States; ^3^Department of Anesthesiology, Louisiana State University Health Shreveport, Shreveport, LA, United States; ^4^Department of Pharmacy Practice, Thomas J. Long School of Pharmacy and Health Sciences, University of the Pacific, Stockton, CA, United States; ^5^Department of Obstetrics and Gynecology, Louisiana State University Health Shreveport, Shreveport, LA, United States; ^6^University of Arizona College of Medicine-Phoenix, Phoenix, AZ, United States; ^7^Department of Anesthesiology, Creighton University School of Medicine, Omaha, NE, United States; ^8^Valley Anesthesiology and Pain Consultants—Envision Physician Services, Phoenix, AZ, United States; ^9^Southcoast Health, Southcoast Physicians Group Pain Medicine, Wareham, MA, United States

**Keywords:** postpartum depression, postpartum mood disorder, brexanolone, GABA, allopregnanolone

## Abstract

Postpartum depression (PPD) is one of the three major categories on the spectrum of postpartum psychiatric syndromes. Postpartum psychiatric syndromes are classified as either postpartum blues, postpartum depression, or postpartum psychosis. Postpartum depression is important to recognize clinically because of the effect it can have on the mother-child bond. The neurosteroid allopregnanolone, a progesterone derivative, is important for its role in positively modulating GABA_A_ receptors. GABA-mediated signaling has been previously implicated in major depressive disorder. Allopregnanolone-mediated signaling has been identified as an important therapeutic target. Treatment with an allopregnanolone-analog, brexanolone, has been shown to improve depression scores in trials for the treatment of PPD. Brexanolone is a positive allosteric modulator of GABA_A_ and is the first drug approved by the FDA to treat postpartum depression. Brexanolone enhances the inhibitory effects of GABA_A_, restores dysfunctional GABA_A_ transmembrane channels, and mimics a naturally produced progesterone metabolite that fluctuates during pregnancy and postpartum. One open-label study and two phase two studies have some significant reduction in HAM-D scores after treatment and that the effect was still there 30 days post-treatment. Per the data reported, intravenous infusion of brexanolone could be efficacious and safe for the treatment of women suffering from postpartum depression.

## Introduction

Postpartum depression (PPD) can result in mothers' adverse health consequences, disrupt the mother-child relationship, and causes distress to the family unit (1, 2).

PPD is a mood disorder that is defined as a major depressive disorder with postpartum onset. To meet the criteria for a major depressive disorder diagnosis, one must have a depressed mood or loss of interest in activities for at least 2 weeks. Other symptoms such as sleep disturbances, appetite changes, and feelings of worthlessness or guilt may also be present (3, 4). PPD can occur at any time from the onset of birth up to 4 months post-delivery. Many of the same symptoms that new mothers experience during postpartum blues are mirrored during PPD. What separates the two is the severity and duration of symptoms.

PPD can present with anxiety, difficulty sleeping, and weight loss that last longer than 2 weeks and occur daily (1). Risk factors that can lead to the development of PPD include mothers with a history of anxiety or depression, young maternal age, low socioeconomic status, alcohol and substance abuse, or a family history of depression (1, 5, 6). PPD is important to recognize clinically because of the effect it can have on the mother-child bond. If not recognized early, it can result in developmental delays in the infant and impair maternal recognition of infant behaviors and cues (1, 2). It can cause new mothers to develop a lack of interest in caring for their child, which may include developing negative feelings toward providing nutrition for their child through breastfeeding (1, 7). Additionally, mothers with PPD are less likely to discuss the difficulties they feel with their healthcare providers, and their children are more likely to have a greater number of emergency department visits (1, 8).

## Pathophysiology/Presentation

The exact mechanism of development of PPD has eluded broad consensus, and it is a major focus of ongoing research. However, it is evident that the pathophysiology of the condition is a consequence of both psychosocial and biological factors. First, discussed is the gabaergic theory of depression, neurobiological differences see in PPD, and then the neurosteroids implicated in the pathogenesis of PPD.

### Gabaergic Theory of Depression

Another theory in the pathogenesis in depression, in general, is γ-aminobutyric acid (GABA) deficits. There is evidence in the research that depression causes alterations in the subunit composition in GABA_A_ subunits of GABA receptors which inhibits GABA neurotransmission (9). Gabaergic transmission is thought to play a part in the control of hippocampal neurogenesis and neural maturation (9), which plays a part in the cognitive and memory deficits that can be seen in depression. GABAergic drugs, such as progabide and fengabine, have shown in animal studies to reverses the behavioral deficits seen in the animal models of depression (10). GABA is also thought to play a role in the mediation of stress which is increased in depression and could play a part in PPD (10). Without GABA mediation, the effects of stress on cognition is increased.

### Neurobiological Presentation

The role of neurobiological changes in the development of PPD has also been a topic of an ongoing investigation, primarily through the use of functional magnetic resonance imaging (fMRI) studies in human and animal models. Activation fMRI studies demonstrated that depressed mothers exhibited significantly less activation in brain networks related to normative parenting in response to their infant's emotional facial and expressions and the sound of their infant crying (11, 12). Resting-state fMRI studies have also shown that PPD is associated with disrupted connectivity in the corticocortical and corticolimbic systems, particularly between the amygdala, anterior cingulate cortex, dorsal lateral prefrontal cortex, and the hippocampus (13, 14). Previous analyses of magnetic resonance imaging studies have shown that the neurobiological presentation of postpartum depression has unique distinctions from that of major depressive disorder, although there are varied interpretations of the relative significance of these distinctions (15, 16).

### Neurosteroidal Regulation

Reproductive hormones directly regulate the biological systems that have been previously implicated in the development of major depression, are involved in mediating emotion processing and cognition, and modulate the neural reward system (17, 18). Additionally, research performed by Ahokas et al. (19) and Gregoire et al. (20) found that estradiol was an effective treatment for women with PPD in reducing depressive symptoms, suggesting dysregulation of the endocrine system as a potential etiology. A study of 25 women followed every 3 days for the first 6 weeks postpartum found that there was a weak association between postpartum depression and the magnitude of the change of progesterone (21). However, multiple studies examining estrogen and progesterone levels and postnatal depressive symptoms have failed to yield a conclusive association between ovarian hormones and the development of PPD (22–24), despite depressive symptom onset being temporally coincident with the rapid fluctuations in ovarian hormones that occur during delivery (23, 25).

Nevertheless, environmental stressors and biological conditions have been identified as confounding factors in previous studies. Controlling for these variables, Bloch et al. (25) conducted a study that showed euthymic women with a history of PPD experienced significant mood dysregulation after treatment and subsequent withdrawal of estradiol and progesterone, while women without a history of PPD did not experience mood dysregulation under identical hormonal conditions (25). Notably, this provides support for the proposed hormone-sensitive PPD phenotype, a model in which perinatal fluctuations of estrogen and progesterone act as a powerful stressor for a subpopulation of susceptible women.

Fluctuations in neuroactive steroid hormone metabolites, or neurosteroids, have also been studied as mediators of PPD. The neurosteroid allopregnanolone, a derivative of progesterone, is important for its role in positively modulating GABA_A_ receptors. GABA-mediated signaling has been previously implicated in major depressive disorder (9, 26), and a study done by Deligiannidis et al. (27) demonstrated that decreased GABA levels were associated with increased depression scores in women at risk for postpartum depression. Additional studies have shown that decreased levels of allopregnanolone are associated with increased depressive symptoms in pregnant women (28), and increased allopregnanolone levels have been correlated with a decreased risk of developing PPD (29). As of this writing, there has been no randomized controlled trials looking at progesterone to treat postpartum depression. However, allopregnanolone-mediated signaling has also been identified as an important therapeutic target, and treatment with an allopregnanolone-analog, brexanolone, has been shown to improve depression scores in trials for the treatment of PPD (30).

### Current Treatment of PPD

There are many treatment options available to women who have postpartum depression. If depression is categorized as mild-to-moderate, psychotherapy is recommended as a treatment. Moderate-to-severe postpartum depression treatment includes antidepressant medications in combination with psychotherapy. The consequence of not treating postpartum depression can include increased disease burden and increase risk of repeat postpartum depression in the mother, and developmental delays in children (31).

When evaluating which medication to choose in the treatment of moderate-to-severe postpartum depression, it is crucial to consider if the patient has been responsive to an antidepressant before developing postpartum depression (31). Suppose this medication history is not available or the patient has not been on an antidepressant before this. In that case, first-line effective treatments are SSRIs, like citalopram and sertraline, favorable both for patient response and favorable side effect profile (31).

A special consideration when discussing pharmacologic therapy in postpartum depression is whether mothers will be breastfeeding their child; “although there is not strong evidence that the amount of antidepressant medication in the breastmilk is sufficient to cause harm for the infant, many women prefer to avoid even this small potential risk” (32). Another consideration is the knowledge that pregnant women worry about the stigma of taking medication and the shame of taking antidepressants. This is what can make brexanolone a reasonable treatment option as the patient does not have to receive daily treatment after their initial infusions.

### Brexanolone

**Figure 1:**
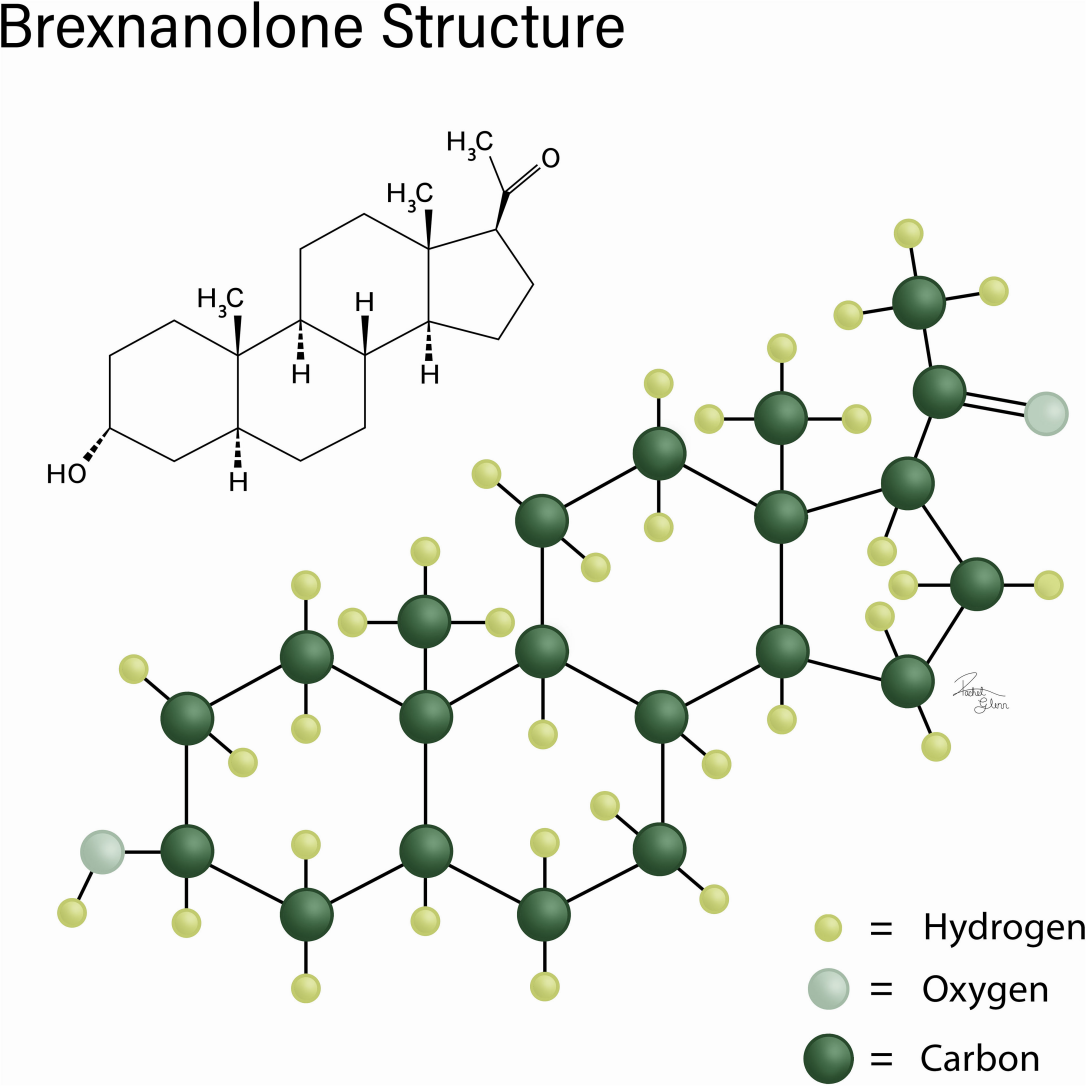
Chemical structure of brexanolone.

Brexanolone is a positive allosteric modulator of GABA_A_ and is the first drug approved by the FDA to treat postpartum depression (33, 34). Brexanolone is only available under a limited program using a risk evaluation and mitigation strategy (REMS). Restricted use of brexanolone was adopted to limit the harmful effects that could possibly occur with brexanolone treatment (35). Before beginning a patient on brexanolone, there are several guidelines, considerations, and possible side effects of which the healthcare providers must make their patients aware. Figure 1 shows the chemical structure of brexanolone.

### Administration

Currently, the only means by which brexanolone is administered is intravenously for a total of 60 h. Before administration, brexanolone must be diluted, and the specific administration requirements of brexanolone requires the patient to be hospitalized at an approved health care facility for the duration of the IV infusion (34, 35). Initial dosing of brexanolone begins at 30 mcg/kg/h for the first 4 h, and then dosage increases to 60 mcg/kg/h from hours 4–24. Dosage is again increased to 90 mcg/kg/hr from hours 24–52 and is subsequently decreased to 60 mcg/kg/hr from hours 52–56 and back to 30 mcg/kg/h for hours 56–60 (35).

### Side Effects and Considerations

Brexanolone has been reported to cause sedation, somnolence, loss of consciousness, and altered mental status. As a result, it is recommended that patients not engage in any activities that require high levels of alertness, such as operating a motor vehicle. Brexanolone should be avoided in patients with end-stage renal disease (eGFR <15/min/1.73 m^2^). Patients with end-stage renal disease can have difficulty clearing betadex sulfobutyl ether sodium, which is a solubilizing agent that could accumulate, causing more damage to the kidneys. Patients with mild or moderate renal impairment don't require any adjustment to the normal dosing of brexanolone (35). Brexanolone can inhibit CYP2C9, which is a cytochrome p450 enzyme that is responsible for the metabolism of certain drugs and compounds. Care should be taken when brexanolone is co-administered with drugs that are metabolized by CYP2C9 (34, 36).

### Mechanism of Action

Brexanolone administration results in a multitude of therapeutic effects that relieve symptoms of postpartum depression. Brexanolone enhances the inhibitory effects of GABA_A_, restores dysfunctional GABA_A_ transmembrane channels, and mimics a naturally produced progesterone metabolite that fluctuates during pregnancy and postpartum.

Brexanolone is a positive allosteric modulator of GABA_A_ receptors (33). GABA is a major inhibitory neurotransmitter that acts in the central nervous system. GABA mediates its effects in the central nervous system through both the GABA_A_ and GABA_B_ receptors. GABA_A_ receptors are made up of five transmembrane units that form a channel that is permeable to chloride. The binding of GABA to GABA_A_ receptors results in an opening of the transmembrane channel, and the influx of chloride results in suppression of neural activity in the brain (37, 38). Brexanolone administration enhances the inhibitory effects that GABA induces when binding to GABA_A_ receptors. Enhancing the inhibitory effects on GABA_A_ receptors causes an acute decrease in anxiety levels and reduces depression symptoms (33, 39). Damage or dysfunction to the transmembrane channels that make up GABA_A_ receptors can result in anxiety and neurodevelopmental disorders (37). Brexanolone can be used therapeutically to restore dysfunctional GABA_A_ receptor activity leading to an alleviation of depression symptoms (34, 40).

Brexanolone structurally mimics allopregnanolone, which is a natural progesterone metabolite that is produced by the corpus luteum and placenta during pregnancy (34, 40). Allopregnanolone acts as a positive allosteric modulator at GABA_A_ receptors, causing an increased inhibition of neural activity in the brain. During pregnancy, allopregnanolone reaches peak levels and falls drastically postpartum. The changes in allopregnanolone levels during pregnancy and postpartum are believed to be related to the symptoms caused by postpartum depression. Brexanolone stabilizes the fluctuations of allopregnanolone levels resulting in an improvement of postpartum depression symptoms (33).

### Pharmacokinetics

Brexanolone exhibits linear, time-independent, and dose-dependent pharmacokinetics in a dosage range of 30 mcg/kg/h to 270 mcg/kg/h (41, 42). It is likely that brexanolone achieves extensive uptake into tissues, which is supported by its estimated volume of distribution being 3 L/kg. Plasma protein binding was also found to be >99%, independent of plasma concentrations after administration (42). Brexanolone also exhibits rapid, dose-independent clearance from plasma, at ~1 L/h/kg, and it has an average terminal half-life of 9 h. In contrast to other drugs that are oxidized by cytochrome P450 (CYP-450) enzymes, brexanolone is metabolized by non-CYP pathways and thus modified to enhance its excretion; brexanolone primarily undergoes keto-reduction, glucuronidation, and sulfation (42, 43).

Although it was determined that CYP2C9 was inhibited by brexanolone, *in vitro* studies demonstrated that brexanolone administration did not affect CYP2C9 substrate pharmacokinetics would thus not require dosage adjustment when paired with CYP2C9 substrate drugs. No significant differences were identified in brexanolone pharmacokinetics in populations with severe renal or hepatic impairment. Still, the use of brexanolone is not recommended in patients with end-stage renal disease due to possible accumulation of the solubilizing agent used in the drug's IV formulation (41, 42). The excretion of brexanolone was characterized by radiolabeling, and it was determined that 47% of the administered radiolabeled drug was recovered in feces, while 42% was recovered in urine (42).

### Pharmacodynamics

Brexanolone exhibits a modulating effect on GABA-mediated signaling. In mammalian cells expressing α_1_β_2_γ_2_, α_4_β_3_δ, and α_6_β_3_δ receptor subunits, GABA-mediated currents from recombinant human GABA_A_ receptors were potentiated by brexanolone (42). However, no pharmacodynamic studies have been conducted in humans to examine the effect of brexanolone administration on GABA_A_ receptor function. A dose-response has been associated with increased sedation at subtherapeutic doses, but a similar relationship has not been identified within therapeutic dose ranges. Concomitant use of brexanolone with benzodiazepines and oral antidepressants were also associated with additive risks in the incidence of sedation-related events, potentially due to pharmacodynamic interactions with the central nervous system and the GABA_A_ receptor (41).

Additionally, exposure-response relationships and the time course of the drug's pharmacodynamics remain unknown. The effects of brexanolone on cardiac electrophysiology were studied in a QT study of 30 healthy adults. It was found that brexanolone did not prolong the QT interval at 1.9-times the highest recommended infusion exposure, 90 mcg/kg/h (42).

### Clinical Studies: Safety and Efficacy

While more research needs to be created in the evaluation of brexanolone as a treatment for postpartum depression, current research on the topic provides promising results. One open-label study involving women 18–45 years of age were enrolled in the study (44). Four women with a Hamilton Rating Scale for Depression (HAM-D) score >20 were admitted 14–20 days following the birth of their child. These participants received brexanolone that was up-titrated for 12 h to a 3^rd^ trimester allopregnanolone level, levels were maintained for 36 h, and then this was followed by a 12-h down-taper. Patients were monitored for one and a half days post-infusion and had a follow-up on days 11 and 34. This study reported a mean initial HAM-D score of 26.5 ± 4.1 at time point 0 before treatment with brexanolone. Brexanolone treatment resulted in a significant change from the beginning of the trial improving to HAM-D mean total scores of 1.8 ± 1.5 at hour 60, that is, at the brexanolone infusion's completion. At hour 84, which was after 24 h of observation following the brexanolone treatment completion, the HAM-D mean score was 5.3 ± 2.9 (44).

This significant change in postpartum depression HAM-D scores following treatment with brexanolone was mirrored in a second study (30). The study was a phase 2 trial of brexanolone for the treatment of severe postpartum depression (HAM-D score >26) involving 21 women in a double-blind, randomized, placebo-controlled trial across four U.S. hospitals. Patients were randomized to receive either placebo (*n* = 11) or brexanolone (*n* = 10) intravenously, continuously for 60 h, after which patients were followed for 30 days. Patients in this study were evaluated at time point 0, before brexanolone infusion, and at the 60-h mark. Comparing those two-time points, patients who received brexanolone had a reduction of 21.0 points from the original HAM-D score (standard error 2.9) vs. the placebo score decrease of 8.8 points (standard error 2.8). These changes from the original HAM-D score were maintained throughout the 30-day follow-up window (30).

Of note, both of the studies resulted in several patients improving to the point of their HAM-D score being ≤7, which is considered remission from postpartum depression. Kanes et al. reported that this was the case for four out of four patients at the 24-h mark following brexanolone infusion (hour mark 84 of the trial) (39). The researchers expanded upon these results, showing remission from postpartum depression was observed in 7 of the 10 women receiving brexanolone treatment and that this effect was maintained until the end of the 30-day follow-up period; comparatively, remission was achieved in only 2 of the 11 placebo-treated patients at 30-day follow-up following their treatment (30).

Kanes, Colquhoun, Gunduz-Bruce, et al. reported 4 out of 10 brexanolone intravenous infusion patients experienced side effects as compared to 8 out of 11 in the placebo group. No death or serious adverse events occurred in brexanolone-treated or placebo study participants. The side effects most reported by participants included: dizziness (*n* = 2 for brexanolone group and *n* = 3 for placebo), somnolence (*n* = 2 for brexanolone and *n* = 0 for placebo groups), sedation (*n* = 1 in brexanolone group vs. *n* = 0 in placebo). Additionally, a moderate adverse event did occur in the brexanolone group (sinus tachycardia), which did not occur in the placebo group (30).

Kanes et al. (30) had all four involved patients develop adverse effects; Sedation was reported by two patients, and other events that were reported included fusion site discomfort, infusion site erythema, infusion site pain, rash, thyroid-stimulating hormone increase, dizziness, flushing, and oropharyngeal pain. These effects were deemed to be mild-to-moderate, and most were self-limited. Three of the four participants had their brexanolone infusion dose-adjusted due to the occurrence of adverse effects, and no participants discontinued due to adverse events (30).

There were two additional double-blind, randomized, placebo-controlled trials with brexanolone, both of which showed clinically significant improvement in patients the treatment of postpartum depression. In study 1, women 18–45 years of age with a Hamilton Rating Scale for Depression (HAM-D) score of >26 were randomly assigned 1:1:1 to receive intravenous brexanolone 90 μg/kg/h, intravenous brexanolone 60 μg/kg/h, or placebo. Each treatment was administered for 60 h, and the patients were followed for a 30-day period. 138 total participants were enrolled, *n* = 45 in the 90 μg/kg/h treatment group, *n* = 47 in the 60 μg/kg/h treatment group, and *n* =4 6 in the placebo group. The mean change from baseline HAM-D score at the 60-h mark was decreased 19.5 (standard error 1.2) in the 60 microgram group, decreased by 17.7 (standard error 1.2) with the 90 microgram group, and 14.6 (standard error 0.8) in the placebo group (36).

In study 2 by the same researchers, women 18–45 years of age with a HAM-D score of 20–25 were randomly assigned in a 1:1 fashion for either brexanolone 90 μg/kg/h or placebo for a total of 60 h. In study 2, 108 total participants enrolled, who were split evenly between treatment and placebo groups (*n* = 54 and *n* = 54, respectively). The mean change from baseline HAM-D score at the 60-h mark was decreased by 14.6 (standard error 0.8) vs. 12.1 (standard error 0.8) in the placebo group. This second study also highlights brexanolone's efficacy in treating postpartum depression (36).

Throughout both studies performed by Metzer-Brody et al. (36), the most common adverse effects were: headache, dizziness, and somnolence. Study 1 had a notable side effect (*n* = 1) of suicidal ideation and attempted overdose in one participant of the 60-μg group. Study 2 had two serious adverse events occur in one participant of the treatment group (syncope and altered state of consciousness). A small number of patients in both studies reported nausea or injection site pain, but this was not significantly different from placebo. Fatigue was another common side effect in patients treated with brexanolone infusion, which did not occur in placebo patients of either study 1 or study 2. Death did not occur in any patients in either study (36).

There was an analysis of 26 studies resulted in 6 similar studies comparing brexanolone and SSRIs in regard to the HAM-D score. Matching-adjusted indirect comparisons were performed for brexanolone treatment vs. placebo, SSRI treatment vs. placebo, and brexanolone treatment vs. SSRI treatment, and the data were reported in terms of change from baseline (45). Change from baseline was greater in women with postpartum depression being treated with brexanolone as compared with women being treated with SSRIs. Change from baseline between brexanolone and SSRIs were 12.79 (8.04–17.53) on day 3, 5.87 (–1.62 to 13.37) in week 4, and 0.97 (–6.35 to 8.30) upon final observation for the HAM-D in both patient and clinician-reported outcomes. The data reviewed show that brexanolone is effective to treat postpartum depression, though further research is warranted (45).

Throughout both studies performed by Metzer-Brody et al., the most common adverse effects were headache, dizziness, and somnolence. Study 1 had a notable side effect (*n* = 1) of suicidal ideation and attempted overdose in one participant of the 60-microgram group. Study 2 had two serious adverse events occur in one participant of the treatment group (syncope and altered state of consciousness). A small number of patients in both studies reported nausea or injection site pain, but this was not significantly different from placebo. Fatigue was another common side effect in patients treated with brexanolone infusion, which did not occur in placebo patients of either study 1 or study 2. Death did not occur in any patients in either study (36). Table 1 summarizes the studies discussed in this section.

**Table 1:** Safety and efficacy of brexanolone in treating postpartum depression in adults.

**References**	**Groups studied and intervention**	**Results and findings**	**Conclusions**
Kanes et al. (30)	Phase 2 study of brexanolone for the treatment of severe postpartum depression (HAM-D score >26) involving 21 women in a double-blind, randomized, placebo-controlled trial across four U.S. hospitals. Patients were randomized to receive either placebo (*n* = 11) or brexanolone (*n* = 10) intravenously, continuously for 60 h, after which patients were followed for 30 days	Results were quantified in terms of the Hamilton Rating Scale for Depression (HAM-D). At the 60-h mark, patients who received brexanolone had a deviation of 21.0 points from original HAM-D score (standard error 2.9) vs. the placebo score change of 8.8 points (standard error 2.8). Remission from postpartum depression (HAM-D score <7) was observed in 7 of the 10 brexanolone treatment, and this effect was maintained until the end of the 30-day follow-up period; this was the case with two of eleven placebo-treated patients at 30-day follow-up.4 out of 10 brexanolone patients experienced side effects as compared to 8 out of 11 in the placebo group. While no death or serious adverse events occurred, side effects most reported included: dizziness (*n* = 2 for brexanolone group and *n* = 3 for placebo), somnolence (*n* = 2 for brexanolone and *n* = 0 for placebo groups), sedation (*N* = 1 in brexanolone group vs. *n* = 0 in placebo) and a moderate adverse event did occur in the brexanolone group (sinus tachycardia).	Brexanolone results in clinically significant reduction in postpartum depression, and is well-tolerated by postpartum women.
Meltzer-Brody et al. (36)	Two double-blind, randomized, placebo-controlled trials were performed with brexanolone and discussed in this literature. In study 1, women 18–45 year of age with a Hamilton Rating Scale for Depression (HAM-D) score of >26 were randomly assigned 1:1:1 to receive intravenous brexanolone 90 μg/kg/h, intravenous brexanolone 60 μg/kg/h, or placebo. Each treatment was administered for 60 h, and the patients were followed for a 30-day period.	In study 1, 138 total participants were enrolled, *n* = 45 in the 90 microgram/kilogram/h group, *n* = 47 in the 60 μg/kg/h group, and *n* = 46 in the placebo group. Mean change from baseline (HAM-D) score at the 60-h mark was: decreased 19.5 (standard error 1.2) in the 60 μg group, decreased by 17.7 (standard error 1.2) with the 90 μg group, and 14.6 (standard error 0.8) in placebo group. Nineteen of the participants receiving 60 μg brexanolone, 22 of the 90 μg brexanolone group, and 22 of the placebo group experienced adverse events in study 1.	Brexanolone injection has potential as a novel therapeutic option for postpartum depression, resulting in clinically significant reductions in HAM-D score.
Kanes et al. (30)	Open-label proof-of-concept study involving women 18-45 years of age were enrolled in the study. Four women with a Hamilton Rating Scale for Depression (HAM-D) score >20 were admitted 14–20 days following the birth of their child. These participants received a level of brexanolone up-titrated for 12 h to a third trimester allopregnanolone level, and maintained at that level for 36 h, which was followed by a 12 h down-taper. Patients were monitored for one and a half days post infusion, and had follow-up on days 11 and 34.	Mean initial HAM-D score was 26.5 ± 4.1 at the beginning of the trial. At hour 60, HAM-D mean total score was 1.8 ± 1.5. At hour 84, the HAM-D mean score was 5.3 ± 2.9. From 24 h onward, all patients (*n* = 4) had total HAM-D scores of ≤7, indicating a remission from postpartum depression symptoms. Dose adjustments were required due to incidence in most participants (*n* = 3), but no serious adverse event occurred throughout this trial.	Allopregnanolone is supported as a player in the pathophysiology of postpartum depression, and brexanolone may play a therapeutic effect in the treatment of this condition.
Cooper et al. (45)	Analysis of 26 studies resulted in 6 studies comparing brexanolone and SSRI's in regard to the Hamilton Rating Scale for Depression (HAM-D) score. Matching-adjusted indirect comparisons were performed for brexanolone vs. placebo, SSRI vs. placebo, and brexanolone vs. SSRI, and data were reported in terms of change from baseline	Change from baseline was greater in women with postpartum depression being treated with brexanolone as compared with women being treated with SSRI's. change from baseline between brexanolone and SSRIs were 12.79 (8.04–17.53) [day 3], 5.87 (−1.62 to 13.37) [week 4] and 0.97 (−6.35 to 8.30) [last observation] for the HAM-D in both patient and clinician-reported outcomes	Brexanolone provides clinically similar or improved outcomes in treating women with postpartum depression

## Conclusion

PPD is a depressive disorder that occurs when depression lasts longer than 2 weeks after birth. It is associated with multiple risk factors, including personal history, family history, and some social stresses such as relationship problems, unplanned pregnancy, and having no support from the infant's father. PPD, if left untreated, can cause suffering in the mother, more stress in the family, and possible developmental problems in the infant. For new mothers, asking for help can be a daunting decision, and taking medication on a daily basis can be unwanted. Brexanolone offers a solution for severe PPD. Brexanolone is sold under the brand name of Zulresso, and the cost is around $34,000 dollars per patient. It is the first drug that was FDA approved specifically for PPD. Brexanolone mimics naturally produced progesterone and is thought to act on the GABA_A_ receptors. This is a groundbreaking treatment as it targets the signaling thought to be a deficit in hormone-sensitive postpartum depression as well in the gabaergic hypothesis of depression. It is also an infusion that can free patients from having to take a medication daily which may help in cases where there is a stigma against using daily medications for depression. It is also the first medication that is specifically FDA-approved to treat PPD. In studies, brexanolone has significantly decreased HAM-D scores after the 60-h infusion, and that effect was still seen 30 days post-treatment. Per the data reported, intravenous infusion of brexanolone could be efficacious but also safe for treatment of women suffering from postpartum depression. More studies should be performed to further delineate brexanolone's ability treatment PPD.

## Author Contributions

AE was responsible for the design, literature review, editing, and writing of the manuscript. KL, AKas, and GH were also involved in writing. ADK, AKay, AO, JM, PB, DL, OV, and IU were responsible for editing. All authors listed have made a substantial, direct and intellectual contribution to the work, and approved it for publication.

## Conflict of Interest

The authors declare that the research was conducted in the absence of any commercial or financial relationships that could be construed as a potential conflict of interest.

## Publisher's Note

All claims expressed in this article are solely those of the authors and do not necessarily represent those of their affiliated organizations, or those of the publisher, the editors and the reviewers. Any product that may be evaluated in this article, or claim that may be made by its manufacturer, is not guaranteed or endorsed by the publisher.
